# Gastroprotective effect of phytoncide extract from *Pinus koraiensis* pinecone in *Helicobacter pylori* infection

**DOI:** 10.1038/s41598-020-66603-8

**Published:** 2020-06-12

**Authors:** Se-eun Kim, Azra Memon, Bae Yong Kim, Hyelin Jeon, Woon Kyu Lee, Se Chan Kang

**Affiliations:** 1 Research Institute, Phyrus Co., LTD., Danyang-gun, 27000 Chungcheongbuk-do South Korea; 2 Department of Oriental Medicine Biotechnology, College of Life Sciences, Kyung Hee University, Yongin-si, 17104 Gyeonggi-do Korea; 3 Department of Biomedical Sciences, School of Medicine, Inha University, Incheon, 22212 South Korea

**Keywords:** Gastrointestinal models, Gastroenteritis

## Abstract

For centuries, herbs have been used by traditional therapists around the world to treat gastrointestinal tract disorders, such as gastritis. We hypothesized that the anti-*Helicobacter pylori* properties of phytoncide, which is extracted from pinecone waste, would facilitate use as a natural gastroprotective product to treat gastrointestinal tract disorders. Thus, we investigated *in vitro* antibacterial efficacy against *H. pylori* by agar diffusion assay. To determine the gastroprotective properties of phytoncide, we conducted hematoxylin and eosin staining, performed assays for the detection of the cytotoxin gene, and evaluated pro-inflammatory cytokine expression in *H. pylori*-infected C57BL/6 mice. Phytoncide significantly inhibited the survival of *H. pylor*i in the gastrointestinal system of C57BL/6 mice. Reduction of gastric severity in *H. pylori*-infected mice was associated with reductions in the expression levels of pro-inflammatory cytokines in the gastric mucosa, and of the cytotoxin CagA gene in phytoncide treated groups (*P* < 0.05 and *P* < 0.01). In conclusion, phytoncide significantly inhibited the growth of *H. pylori* in gastro tissue, possibly due to the abundant α-pinene present in the phytoncide as detected by HPLC analysis. Further studies are needed to validate our findings, but we suggest that phytoncide has the potential to be used as a natural ingredient in anti-*H. pylori* products.

## Introduction

*Helicobacter pylori* bacteria are spread worldwide, causing chronic gastritis and affecting more than half of the world’s population, which is of great interest worldwide. When infected with *H. pylori*, it can also lead to peptic ulcer and gastric cancer^1^. In 1994, the World Health Organization (WHO) categorized *H. pylori* as a ‘definite biological carcinogen’^2^. In the last 20 years, treatment for *H. pylori* infections has mainly been based on multiple drugs, such as metronidazole, amoxicillin, furazolidone, tetracycline, and clarithromycin, administered with proton pump inhibitors (PPIs) or bismuth^3^. Treatment for *H. pylori*, which is currently used, is universally effective but may fail primarily due to antimicrobial resistance and patient non-adherence. Therefore, there have been ongoing efforts to develop new drugs to fight this bacterium.

Plants have been used as a medicinal herb to treat diseases for a longtime. Recently, interest in alternative remedies including plant extracts and/or plant-derived substances identified for anti-*H. pylori* efficacy is increasing^4^. A variety of plant species can synthesize substances that show antibacterial activity *in vitro*^5–8^ and *in vivo*^9,10^. In Brazil, the general Brazilian diet and folk remedy have been used to treat gastrointestinal diseases and have been shown to inhibit *H. pylori in vitro* growth^11^. Licorice root, the *Glycyrrhiza glabra* L., has been used for the treatment of *H. pylori-*related stomach ulcers^12^. Some plant extracts that have therapeutic effects can stimulate the immune system to improve resistance to infectious and inflammatory diseases. A team of Mahady *et al*., screened methanol extracts of 24 plant species to determine their inhibitory effect on 15 strains of *H. pylori* bacteria by *in vitro* analysis. As a result, it was found that the most effective extract was an extract of *Myristica fragrans* (seed), which exhibited a minimum inhibitory concentration of 12.5 μg/mL^13^. In addition, other researchers from Bhamarapravati *et al*. investigated the effects of traditional Thai medicine on *H. pylori* 15 strains for the treatment of gastrointestinal illness^14^. The conventional folk remedial use of plants used to treat gastric infections may be due to the antibacterial efficacy of their extracts against *H. pylori*. Therefore, some medicinal plants that have been evaluated for anti-*H. pylori* bacterium activity may be more powerful and less riskiness than conventional remedies, with treatability for gastrointestinal diseases of *H. pylori* origin^15^.

Phytoncide is an antimicrobial volatile organic compound derived from plants that are enriched in terpenoids, alkaloids, and phenylpropanoids. The main ingredients are monoterpenoids, which include α-Pinene, myrcene, and careen^16–18^. According to previous studies, phytoncide has various pharmacological effects and its efficacy includes antioxidants, immune stimulation, anti-cancer, and anti-inflammatory activities^19,20^. We expected to be able to regulate *H. pylori*-induced gastritis through the anti-inflammatory effect of phytoncide and proceeded with this study. We evaluated whether phytoncide inhibits the production of *H. pylori* antibodies and thereby weakens the associated inflammatory response.

## Results

### Inhibition of *H. pylori* growth by phytoncide

**Figure 1:**
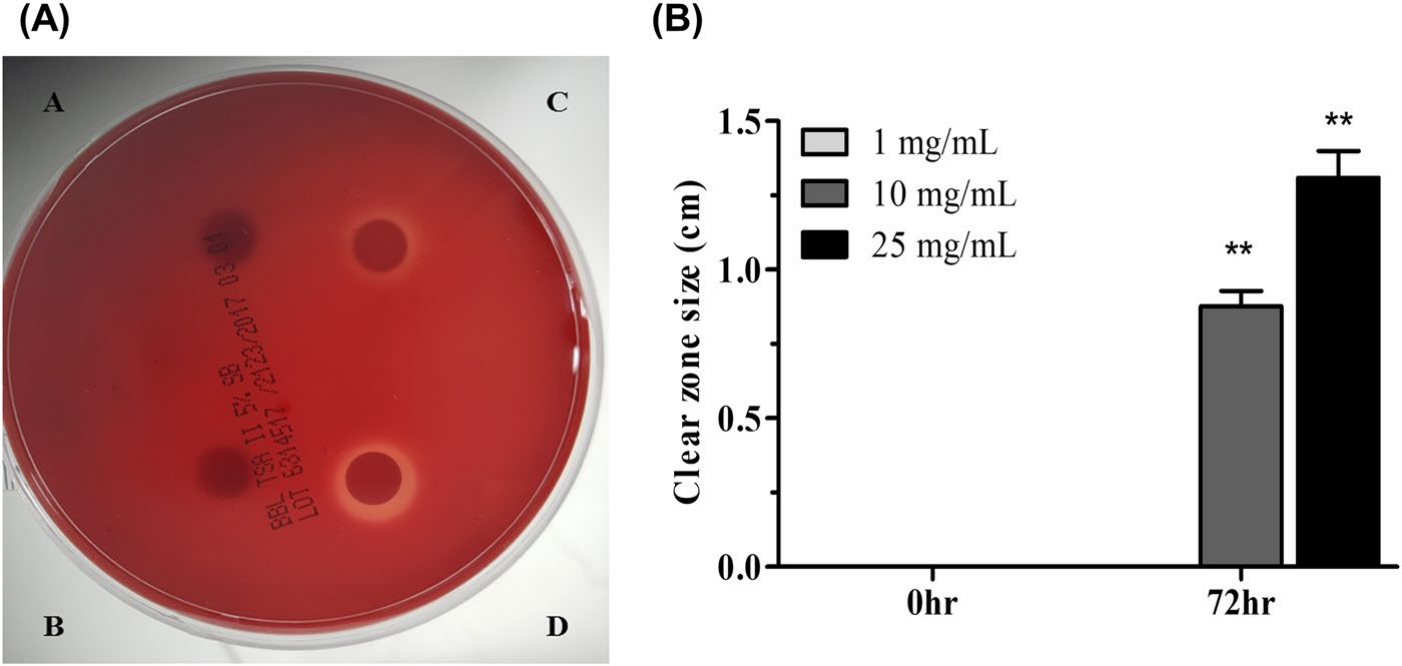
Inhibitory effect of phytoncide against the growth of *Helicobacter pylori*. (**A**) Images represent *H. pylori* inhibition efficacy tests (**A**), Control (DW)); (**B**), 1 mg/mL phytoncide treatment; (**C**), 10 mg/mL phytoncide treatment; (**D**), 25 mg/mL phytoncide treatment). (**B**) Graph of the inhibitory effect of phytoncide against *H. pylori* growth. The diameter of each clear zone was measured. Representative data are expressed as mean ± SEM of three different experiments (*n* = 3) for each group. ^**^p < 0.01 compared to control.

Anti-bacterial effect of phytoncide *in vitro* was first confirmed. To investigate the inhibitory effects of phytoncide against *H. pylori* growth and development, phytoncide concentrations of 1, 10, and 25 mg/mL were used and estimated after 72 h. As shown in Fig. 1, treatment with 1 mg/mL of phytoncide did not show any inhibitive effects after 72 h of cultivation, while 10 mg/mL of phytoncide showed 0.87 cm inhibition and 25 mg/mL showed 1.31 cm inhibition (diameter of the clear zone). In this experiment, have a justification of the *in vivo* experiment start was obtained by confirming the antimicrobial activity of phytoncide at the *in vitro* level.

### Regular symptoms in experiment mice

**Figure 2:**
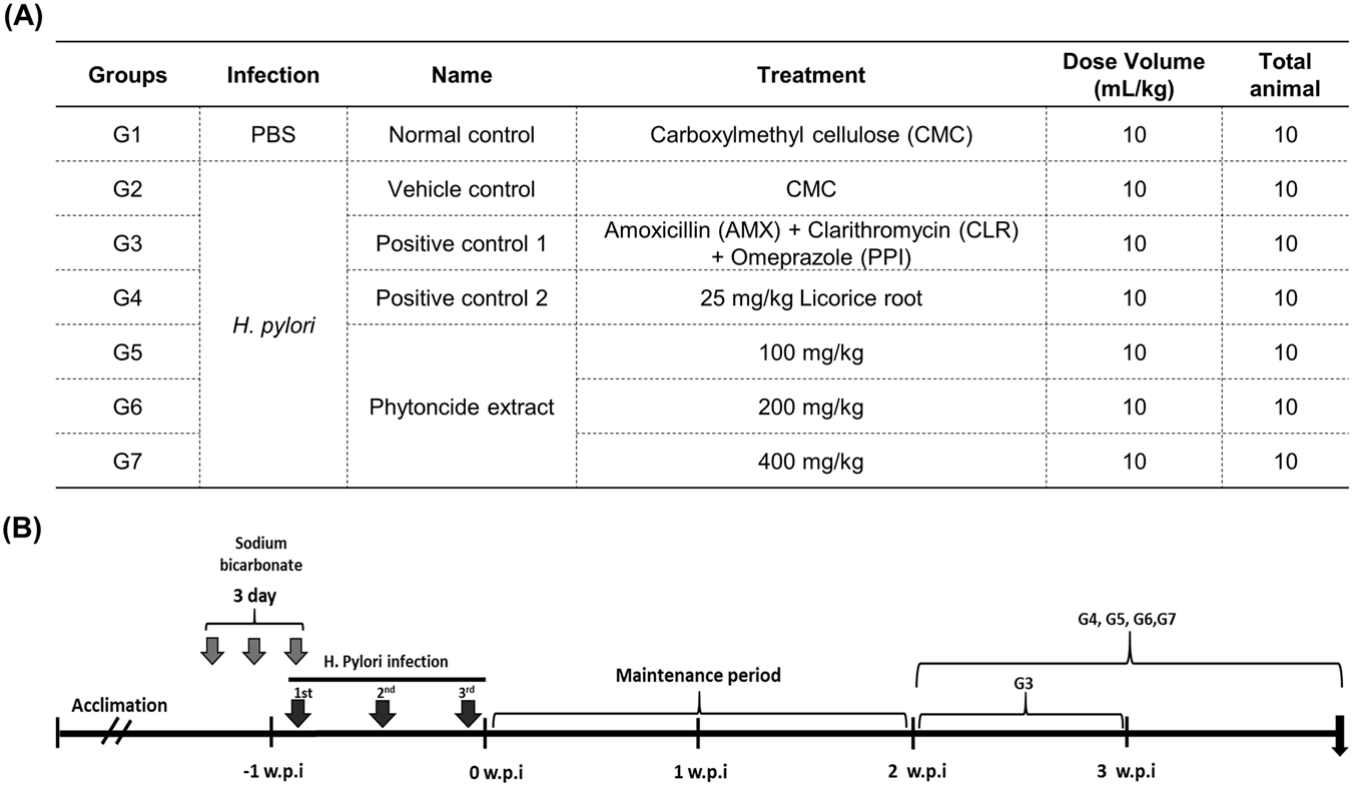
Design of experiments with C57BL/6 mice: Effects of phytoncide against *Helicobacter pylori* infection in the gastrointestinal system. (**A**) Animal experimental groups. G1: Normal control group (CMC, *n* = 10), G2: Vehicle control group (*H. pylori* + CMC, *n* = 10), G3: Positive control group 1 (*H. pylori* + 14.25 mg AMX + 7.15 mg/kg CLR + 400 μM/kg Omeprazole, *n* = 10), G4: Positive control group 2 (*H. pylori* + 25 mg/kg Licorice root extract, *n* = 10), G5: Phytoncide group 1 (*H. pylori* + 100 mg/kg Phytoncide, *n* = 10), G6: Phytoncide group 2 (*H. pylori* + 200 mg/kg Phytoncide, *n* = 10), G7: Phytoncide group 3 (*H. pylori* + 400 mg/kg Phytoncide, *n* = 10). (**B**) Time schedule for animal experiments.

The grouping for the animal experiment was as shown in Fig. 2A, and the experiment progressed as shown in Fig. 2B. There were no special symptoms were observed among experimental mice during the study. There were no significant differences in bodyweight or food and water consumption between the groups throughout the experiment (data are not shown).

### Effect of phytoncide against the *H. pylori* IgG antibodies in the blood

**Figure 3:**
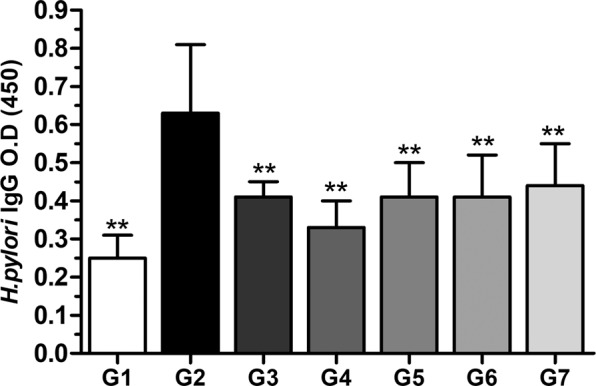
Effect of phytoncide on *Helicobacter pylori* antibody (IgG) in serum. Representative data are expressed as mean ± SEM (*n* = 10) for each group. ^**^p < 0.01 compared to vehicle control group.

We collected blood samples two weeks after infection to determine whether *H. pylori* infection was evident and whether the development of antibodies was reduced by phytoncide. Therefore, blood samples collected from experimental mice were used to measure IgG antibodies of *H. pylori* and the average mean and standard error of the mean (SEM) was calculated. As shown in Fig. 3, in the vehicle control group G2 (*H. pylori* infection, disease) compared to G1 (normal control), *H. pylori* antibodies increased by 152.0%, which confirms the presence of inflammation (*P* < 0.01). In treatment groups G3 (positive control group 1: Amoxicillin (AMX) + Clarithromycin (CLR) + Omeprazole (PPI)) and G4 (positive control group 2: licorice root extract) antibodies decreased significantly (*P* < 0.01) compared to G2, by 34.9% and 47.6%, respectively. In phytoncide groups G5 (100 mg/kg), G6 (200 mg/kg), and G7 (400 mg/kg), antibodies decreased by 34.9%, 34.9%, and 30.2% compared to G2, respectively (*P* < 0.01). The IgG antibodies in phytoncide-provided groups (G5, G6, and G7) almost uniformly decreased compared to G3 (positive control group 1: AMX + CLR + PPI) (Fig. 3). As a result, these results indicate that phytoncide has an anti-inflammatory activity against *H. pylori* as much as a drug cocktail. There was no dose-dependent result among the phytoncide-provided groups, which was expected to be due to the large error between individuals in the same group.

### CLO test

**Figure 4:**
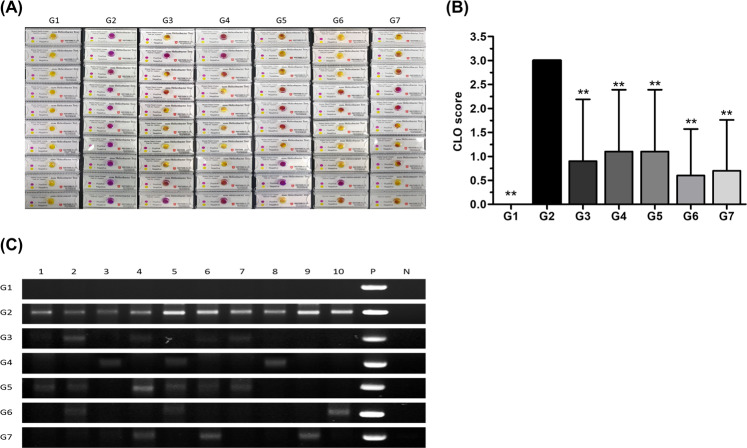
Antibacterial effect of phytoncide against *Helicobacter pylori* according to CLO tests and CagA gene expression. (**A**) The individual values of rapid urease CLO tests in gastric tissue. (**B**) Graph of rapid urease CLO scores. (**C**) Effect of phytoncide on polymerase chain reaction for CagA cytotoxin. Full-length gels are presented in Supplementary Figure S1. Representative data are expressed as mean ± SEM (*n* = 10) for each group. ^**^p < 0.01 compared to the vehicle control group. P stands for positive and N stands for negative.

The *Campylobacter*-like organism (CLO) test is a rapid diagnostic test to diagnose *H. pylori* infection. In the kit, the color gradually changes from yellow to red in the presence of the urease enzyme of *H. pylori*. As shown in Fig. 4A,B, rapid degradation of the CLO element indicated that G2 was significantly higher in urease than G1 and that it was therefore infected with *H. pylori* (*P* < 0.01). The G3 and G4, on the other hand, maintained levels of 30% compared to G2, confirming that each positive control worked properly (*P* < 0.01). Meanwhile, G5, G6, and G7 were almost 38%, 21%, and 23%, respectively, compared to G2 (*P* < 0.01). The CLO scores were lowest in G6 and G7, these groups were treated with 200 and 400 mg/kg phytoncide. We thus confirmed that phytoncide was effective for suppressing infection of *H. pylori*.

### Detection of the cytotoxin gene in *H. pylori* DNA by PCR

In order to confirm the degree of infection of CagA-positive *H. pylori*, CagA gene expression of each experimental animal was measured using the polymerase chain reaction (PCR) technique. The agarose gel electrophoresis image shown in Fig. 4C is the result of visualizing the PCR amplification products for CagA in all experimental animals. A strong band was detected in the *H. pylori*-positive group (G2), indicating a self-cure rate of zero. On the other hand, the treatment rates in positive groups G3 and G4 were 60% and 70%, respectively (*P* < 0.01). In phytoncide-provided groups G5, G6, and G7, the self-cure rates were 40%, 70%, and 70%, respectively (*P* < 0.01), which were better outcomes than in the positive control groups.

### Macroscopic and histopathological analyses of stomach mucosa

**Figure 5:**
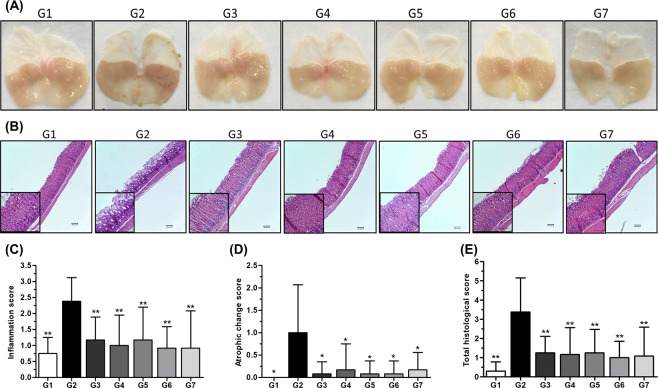
Gastric histology and gastric pathology scores. (**A**) Simple visual observations. (**B**) Hematoxylin and eosin histochemical stain observations. G1, Normal control group; G2, Vehicle control group; G3, Positive control group 1 (14.25 mg/kg AMX + 7.15 mg/kg CLR + 400 μM/kg PPI); G4, Positive control group 2 (25 mg/kg licorice root extract); G5, Phytoncide group 1 (100 mg/kg Phytoncide); G6, Phytoncide group 2 (200 mg/kg Phytoncide); and G7, Phytoncide group 3 (400 mg/kg Phytoncide). The images shown represent the average of each group. (**C**) Inflammation score. (**D**) Atrophic score. (**E**) Total inflammation and atrophic (histological) score. Representative data are expressed as mean ± SEM (*n* = 10) for each group. ^*^p < 0.05 and ^**^p < 0.01 com*p*ared to the vehicle control group.

When a simple visual examination observed the condition of the gastric mucosa, it was confirmed that it has bloodshot in G2 compared to other groups. However, there was no statistically significant difference between the G1 and other experimental groups (Fig. 5A). As shown in Fig. 5B, as a result of histopathological examination, inflammation and cell infiltration and atrophic changes in the gastric mucosa were observed in G2 infected with *H. pylori*. The positive control groups G3 and G4 showed mild damage compared to G2. Phytoncide treatment led to sufficient inhibition of inflammatory processes in groups G5, G6, G7 when compared to the G2. In Fig. 5B, we quantified the changes in the tissues and show the inflammation score (Fig. 5C), atrophic change score (Fig. 5D), and total histological score (Fig. 5E) values in graphs. Compared with G2, the inflammation score and total histological score of the phytoncide showed significant changes with *P* < 0.01. Similar results were observed for the atrophic change score, with a significant change (*P* < 0.05) compared to G2.

### Analysis of cytokine in gastric mucosa

**Figure 6:**
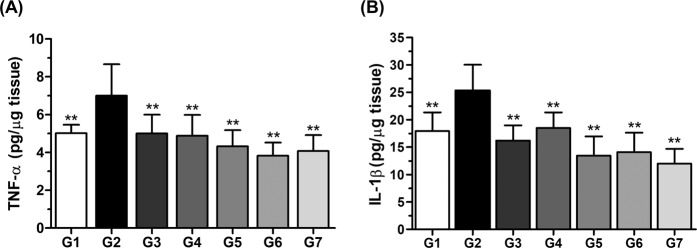
Effect of phytoncide on the expression levels of pro-inflammatory cytokines in *Helicobacter pylori*-infected gastric tissues. (**A**) Effect of phytoncide on TNF-α expression in gastric tissues. (**B**) Effect of phytoncide on IL-1β expression in gastric tissues. Representative data are expressed as mean ± SEM (*n* = 10) for each group. ^**^p < 0.01 compared to the vehicle control group.

The reduction of gastric inflammation severity in *H. pylori*-infected mice was associated with reductions in the expression levels of pro-inflammatory cytokines of gastric mucosae, such as TNF-α and IL-1β^21,22^. Thus, we analyzed TNF-α IL-1β cytokines to confirm inflammatory changes (Fig. 6A,B). Expression of TNF-α increased in the vehicle control group (G2), but was significantly decreased in other experimental groups (*P* < 0.01). Moreover, phytoncide groups (G5, G6, and G7) showed lower expression compared with positive controls (G3 and G4). IL-1β was found to be highly expressed in the gastric mucosa of G2, similar to the results for TNF-α (*P* < 0.01). In addition, the expression of IL-1β was more inhibited by phytoncide treatment (G5, G6, and G7) than in positive control groups (G3 and G4). Inhibition of TNF-α and IL-1β expressions did not significantly differ between groups that received different concentrations of phytoncide.

### HPLC analysis of phytoncide

**Figure 7:**
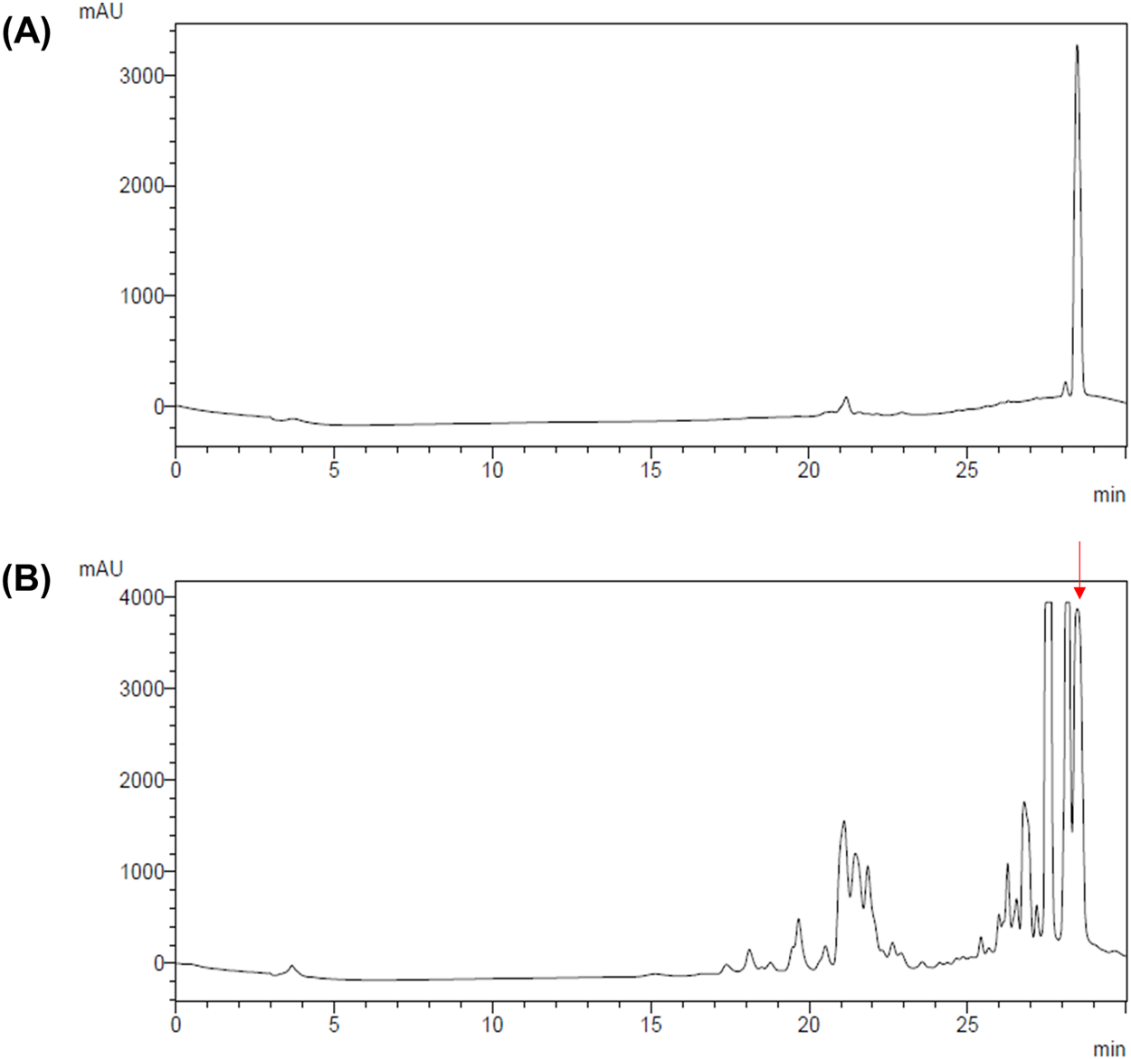
HPLC chromatogram of phytoncide from pinecone waste. (**A**) Standard chromatogram of α-Pinene, (**B**) Chromatogram of 10% phytoncide from pinecone waste.

Through a series of studies, we verified the anti-inflammatory, antimicrobial, and tissue damage inhibitory effects of phytoncide and found that it had a significant effect. However, it was not possible to know which component of the phytoncide is caused by the analytical test. Therefore, we analyzed the chemical profile of phytoncide using GC-MS and identified 9 compounds (data not shown). We analyzed the content of 10% phytoncide by standard α-Pinene (Fig. 7) and determined the content of α-Pinene to be 61.56 mg/g.

## Discussion

We conducted this study to discover natural products that can safely and effectively suppress infections and inflammation of *H. pylori* that can cause gastroenteritis, ulcers, and even cancer in people around the world. We planned to discover materials that have low toxicity and antibacterial and anti-inflammatory properties among natural plants. Pinecone (nut removed) is readily available from nut pine trees that are abundant in Korea, and the phytoncide obtained from pinecone has strong anti-inflammatory effects^23,24^. For this reason, among many natural materials, phytoncide extracted from pinecone waste was selected as research material. First, we conducted an *in vitro* experiment to confirm that phytoncide has antibacterial activity against *H. pylori*, and as a result, confirmed that it has an antibacterial effect at 10 and 25 mg/mL (*P* < 0.01).

Based on the results of this *in vitro* assay, the authors conduct a study it was to confirm the gastroenteritis suppressing efficacy of phytoncide in *H. pylori*-infected 4-week-old C57BL/6 mice. We compared and evaluated the effectiveness of phytoncide by using a positive control as a combination of AMX + CLR + PPI^25^, a drug used against *H. pylori* infection, and Licorice root extract, one of the folk remedies^12^, for *H. pylori*-related stomach inflammations. There are various invasive and noninvasive methods for the detection of *H. pylori* infection. Invasive methods require gastric tissue and endoscopy and including histologic evaluation, bacterial culture, and urease activity testing. However, invasive methods are limited due to the high cost and the possibility of endoscopic and sampling errors due to the non-uniform distribution of *H. pylori* in the stomach^26^. In a previous study, the measurement of IgG demonstrated the highest sensitivity in the *H. pylori* infection^27^. Therefore, we chose a noninvasive method to determine whether or not a successful *H. pylori* infection occurred, and blood samples collected from experimental mice were used to measure IgG antibodies of *H. pylori*. As shown in the results in Fig. 3, the presence of IgG antibodies in the blood increased significantly when infected with *H. pylori*. It was confirmed that the amount of IgG antibody was remarkably decreased in both the positive control group and the experimental group, and phytoncide showed a similar level of efficacy to that of the drug complex G3 regardless of the concentration (*P* < 0.01). Subsequently, the authors continued to evaluate the diagnosis and inhibition of *H. pylori* infection. It was the CLO test and CagA gene expression confirmation. The CLO test shown in Fig. 4A, B is used to confirm the secretion level of the urease enzyme. Urease helps *H. pylori* to survive in an acidic gastric environment during colonization, causing ammonia production, and inducing the cell damage and epithelial inflammation^28^. CagA gene expression used as another evaluation marker was confirmed by PCR analysis (Fig. 4C). *H. pylori* bacteria are characterized as a CagA gene-positive, and this gene was used in that it does not exist in rodents. In the CLO test, the error between individuals in the positive control group and the experimental group was large, but the secretion of urease was significantly reduced. And, it was confirmed that the effect was the best in G6 and G7 provided with phytoncide. In addition, in the CagA gene PCR analysis evaluation, it was confirmed that gene expression was most inhibited in G6 and G7 among the groups that provided phytoncide. These results thus far confirmed that the provision of phytoncide inhibits *H. pylori* bacterial infection.

When the inflammation caused by *H. pylori* infection persists for a long time, atrophy and irregular changes occur on the gastrointestinal surface^29^. Therefore, we evaluated the direct inflammation using the animal’s stomach. The significant difference in the general visual examination was that bleeding of the gastric mucosa was observed only in G2, and no abnormality was found in other groups (Fig. 5A). According to the literature, *H. pylori*, in which is the spiral-shaped bacterium can be identified by hematoxylin and eosin (H&E) staining, and the sensitivity and specificity of H&E staining are 69–93% and 87–90%, respectively^30^. Therefore, H & E staining analysis was performed to observe histological changes and to record inflammation, atrophic change, and total histological score. According to previous researches, *H. pylori*-related chronic gastritis has been to show neutrophil infiltration in the epithelium as well as lymphocyte and plasma cell infiltration^31^. As can be seen from the results of Fig. 5B, our study also showed the atrophy and infiltration of gastrointestinal mucosal cells, irregular cell array, and inflammation by the *H. pylori* infection. These changes were converted into scores and then expressed in graphs, all of which were confirmed to have statistically significant changes compared to G2 (*P* < 0.05). In particular, it was observed in the groups that provided phytoncide that the degree of tissue damage and inflammation was suppressed regardless of the concentration. Lastly, the expression of the proinflammatory cytokine TNF-α and IL-1β was measured that confirm the effect of phytoncide on the reduction of gastric inflammation severity of *H. pylori*-infected mice. This is because *H. pylori*’s gastrointestinal infection is associated with induction of mucosal damage, marked inflammatory response, and high serum levels of TNF-α^32,33^. In the group that provided phytoncide, the expression levels of proinflammatory cytokines were lower than those of the positive controls G3 and G4. However, no concentration-dependent difference of phytoncide was found. This was judged to be due to differences between individuals in the group. We confirmed the strong antibacterial and anti-inflammatory effects of phytoncide on *H. pylori* through a series of results and discovered the potential for medicinal use of it.

The phytoncide was analyzed through HPLC analysis, and as a result, it was confirmed that α-pinene was abundantly present. α-Pinene is highly bioavailable, exhibiting 60% human pulmonary uptake with rapid metabolism or redistribution^34^. According to previous studies, α-Pinene has anti-inflammatory effects via prostaglandin E (PGE)-1^34^ and seems to show anti-microbial activity^35^. Therefore, we decided that the anti-microbial activity of phytoncide may be due to α-Pinene.

In conclusion, histopathological changes, expression of inflammatory cytokines, and expression of CagA gene were decreased in experimental groups treated with phytoncide. Experimental groups that received phytoncide also showed lower antibody levels and higher self-cure rates. These results are thought to be due to a-pinen present in phytoncide, in-depth studies to determine the mechanism of action of phytoncide against *H. pylori* are required. However, we conclude that phytoncide has useful potential as an herbal treatment for gastrointestinal disorders caused by *H. pylori* infection, such as gastric cancer.

## Materials and methods

### Chemicals

Amoxicillin (AMX), clarithromycin (CLR), and omeprazole (PPI) were purchased from Sigma-Aldrich (St. Louis, MO, USA). Licorice root was donated by PHYLUS (Danyang, Chungcheongbuk-do, Korea). A mouse *H. pylori* antibody IgG ELISA kit (Cusabio Biotech Co., Houston, TX, USA) was used to measure anti‐*H. pylori* IgG. Hematoxylin and eosin for staining was purchased from Sigma-Aldrich.

### Preparation of pinecone extract (phytoncide)

Pinecones from *Pinus koraiensis* were provided by INHA University, Incheon, Korea. Dr. Woon Kyu Lee identified the specimens, and a voucher specimen of *P. koraiensis* (voucher # IH-01-035) was deposited in the herbarium of the Department of Biomedical Sciences, School of Medicine, INHA University (Incheon, Korea). Phytoncide extracted from pinecone waste (*P. koraiensis*) was provided by PHYLUS. The phytoncide extraction method was the same as that used by Kim and Lee^36^, and is briefly described as follows. In August 2014, pinecone waste was purchased in Gapyeong (Gyeonggi-Do, Korea). The pinecone waste has 28% water content, however and was reduced to pieces 2–4 cm in size and dried in the shade until it reached 20% water content. One kg of dried cut pinecone was placed in a 5 L round-bottomed flask and the distillation temperature was maintained at 100 ± 3 °C. At the same time, steam was generated and allowed to pass directly to the lower end of the materials for 30 min. The solution obtained by distillation was separated into an oily phase and water phase by differences in specific gravity (cooling water; 16–20 °C). The oily phase (phytoncide) was used in experiments. The yield of extracted phytoncide essential oil was calculated using the following equation:

$${\rm{Yield}}\,{\rm{of}}\,{\rm{essential}}\,{\rm{oil}}\,( \% )=\frac{{\rm{weight}}\,{\rm{of}}\,{\rm{essential}}\,{\rm{oil}}\,({\rm{g}})}{{\rm{Sample}}\,{\rm{weight}}\,({\rm{g}},\,{\rm{pinecone}}\,{\rm{waste}})}\times 100$$

The extracts were then concentrated for 16 h at reduced pressure using a rotary evaporator, and stored at −20 °C until use.

### *H. pylori* bacterium culture

The *H. pylori* strain used in this experiment was obtained from the American Type Culture Collection (ATCC, Manassas, VA, USA) and derived from primary cultured samples. The ATCC #43504 strains were stored at −70 °C in 200 μL of *Brucella* broth (BBL^TM^, Becton Dickinson and Company, Franklin Lakes, NJ, USA) containing 10% fetal bovine serum (FBS, Hyclone, Logan, UT, USA) and supplemented with 10% glycerol (Sigma-Aldrich). Then, 5% sheep blood was added to trypticase soy broth and vaccinated into agar culture medium and incubated under 10% CO_2_, 37 °C, and micro-aerobic conditions for 2–3 days.

### *H. pylori* bacterium growth inhibition assay

After culturing *H. pylori* in BBL trypticase soy agar (TSA) w/5% sheep blood plate, we observed changes after 72 h on a paper disk assay. Test substances (phytoncide) were prepared at concentrations of 1, 10, and 25 mg/mL in carboxylmethyl celluluose (CMC) and treated with 100 μL each on paper discs. *H. pylori* bacteria were cultivated on 3 plates. One CMC-treated paper disc was used as the normal control, and the other three discs were simultaneously treated with phytoncide at different concentrations. After the experiment, the diameter of the clear zone was measured.

### Animal care

Four-week-old-male C57BL/6 mice were purchased from Orient-Bio Corporation (Seongnam, Gyeonggi-do, Korea) and acclimated for 1 week before the experiment. Mice were housed in environments with controlled temperature (22 ± 2 °C) and humidity (53 ± 5%) under 12 h light/dark cycles. During the experiment, regular symptoms and the death rate were observed individually. Cases with special symptoms were recorded. Bodyweight, feeding, drinking, and intake were measured once per week.

### Animal experiment

Seventy pathogen-free C57BL/6 mice were used in this experiment. As shown in Fig. 2A, the animals were divided into six *H. pylori*-inoculated groups and one normal control group, each group containing 10 mice. The experiment was performed in the manner shown in Fig. 2B. To increase the level of *H. pylori* infection, 5% sodium bicarbonate (NaHCO_3_) was given for a total of three days, starting two days before infection to the day of infection. NaHCO_3_ was administered through oral gavage at a dose of 0.2 mL. Except for G1 (normal control), the experimental mice (G2 to G7) received *H. pylori* 5.0 × 10^9^/mL colony-forming units (CFU) by oral gavage at a dose of 0.2 mL 3 times for 2 days (every 12 h). We administered 14.25 mg/kg amoxicillin (AMX) + 7.15 mg/kg clarithromycin (CLR) + 400 μM/kg omeprazole (PPI) mixture, and 25 mg/kg licorice root extract as positive controls, respectively. The G3 orally received an antibiotic mix that was every day for 1 week, after the maintenance period. Meanwhile, licorice root extract (G4) was resuspended in CMC (25 mg/kg) and administered through oral gavage for 2 weeks. Phytoncide was resuspended in CMC by the designated amounts and administered through oral gavage 100, 200, and 400 mg/kg/day for 2 weeks in experimental groups. The experimental procedures and animal care protocols were approved by the Animal Care and Use Committee of National Center of Efficacy Evaluation for the Development of Health Products Targeting Digestive Disorders (NCEED, Incheon, Korea), and conformed to the Guide for the Care and Use of Laboratory Animals of the National Institutes of Health (NIH Publication No. 85-23). Animal experiments were performed in accordance with the current ethical regulations for animal care and use at NCEED (IRB File No. 2018-11-020).

### Infection of mice with *H. pylori* bacteria

It is difficult to achieve stable *H. pylori* colonization within the gastric mucosa of mice, and mice have been acknowledged to be resistant to *H. pylori* infection. A standardized mouse model of *H. pylori* infection with Sydney strain 1 (SS1) provided important information regarding *H. pylori*-related disease, with effects that were very similar to those in humans^37^. Therefore, we infected C57BL/6 mice with Sydney strain 1 (SS1) to estimate the effects of phytoncide in a *H. pylori*-induced animal model of gastritis.

### Visual analysis

After sacrifice, each mouse stomach was removed and cut along the greater curvature towards the duodenum, spread, and then simply visually observed. After visual examination, the stomach was fixed in 10% neutral-buffered formalin (NBF, Sigma-Aldrich), embedded in paraffin, and cut into 4 μm thick sections using a Reichert 200 microtome (Leica, Nussloch, Eisfeld, Germany) for further analysis. H&E staining was performed to examine histological lesions. Data for each sample were collected as follows. Corpus, antrum, and overall inflammatory cells were calculated (neutrophils and mononuclear cells) to determine the level of infiltration and evaluate signs of atrophic gastritis. The resulting values were present as graphs.

### Comparison of *H. pylori* antibodies IgG in blood

The effects of phytoncide against bacterial infection were confirmed by assessing the level of antibody present against *H. pylori* in the blood. Plasma samples were analyzed after the end of the experiment using a mouse *H. pylori* antibody-IgG ELISA kit (Cusabio Biotech Co.) to compare the effects in each group. Experiments that used an assay kit were conducted according to the manufacturer’s instructions.

### Campylobacter-like organism rapid decomposition of elements

Gastric mucosal samples were extracted and CLO aseptically collected as part of the following examination for test reagents (Asan Pharm Co., Ltd., Seoul, Korea). Experiments that used an assay kit were conducted according to the manufacturer’s instructions. When yellow color changed to red after incubation (37 °C, 2 h), cultures were examined and the number of individuals per group was recorded as 100% while the number of cultivated samples of H. pylori bacteriostatic treatment was calculated as follows:

$$\frac{{\rm{Number}}\,{\rm{of}}\,{\rm{samples}}-{\rm{number}}\,{\rm{of}}\,{\rm{cultivated}}\,{\rm{samples}}}{{\rm{Number}}\,{\rm{of}}\,{\rm{samples}}}\times 100$$

Calculating the CLO score: No color badge = 0 score; weak red color = 1; light purple = 2 score; and purple = 3 score. The mean and standard deviation of each group were calculated and compared.

### Detection of the cytotoxin gene in *H. pylori* DNA by PCR

The cytotoxin gene, cytotoxin-associated gene A (CagA), was analyzed by PCR with visualization by agarose gel electrophoresis. After finishing the experiment, genomic DNA was aseptically extracted from the gastric mucosa and a PCT test for *H. pylori* PCR was conducted for a special target and poisonous gene of *H. pylori* – CagA that is not present in human or mice. The PCR target size for the *H. pylori* gene is 298 bp. We used the following primers: 5′-ATA ATG CTA AAT TAG ACA ACT TGA AGC GA-3′ (forward) and 5′-TTA GAA TAA TCA ACA AAC ATC ACG CCA T-3′ (reverse). PCR conditions were as follows: denaturation at 94 °C for 5 min, and then 35 cycles 95 °C for 1 min, 57 °C for 30 s, 72 °C for 30 s, with a final extension step at 72 °C for 10 min.

### Anti-inflammatory efficacy of phytoncide

To measure pro-inflammatory cytokines, gastric mucosal samples were aseptically collected, fragmented into small pieces, and placed in liquid nitrogen. Protein samples were extracted for further analysis using a cell lysis buffer and analyzed using an R&D system (Minneapolis, MN, USA) ELISA kit to evaluate tumor necrosis factor (TNF)-α and interleukin (IL)-1β. Experiments that used an assay kit were conducted according to the manufacturer’s instructions.

### HPLC analysis of phytoncide

α-Pinene was detected at 210 nm by a UV detector using an SHIMADZU LC-20AD series HPLC system (Shimadzu Corporation, Kyoto, Japan). The compound was separated on a SkyPack C 18 column (4.6 × 250 mm, 5 μm) at a column temperature of 40 °C. The solvent system consisted of water (A) and methanol (B) with the following gradient elution program: 0 min 10% B; 10 min 75% B; 15 min 80% B; 17 min 95% B; 18 min 100% B; 20 min 100% B; 25 min 75% B; 27 min 10% B; 30 min 10% B. The flow rate was 1.0 mL/min, and the injection volume of the sample was 10 μL. Detection of compound content in the phytoncide was performed using the external standard method, and α-Pinene (268070, Sigma-Aldrich) was used as the standard stock solutions (12.5, 25, 50, 100, and 200 μg/mL). Water (Fisher Scientific Korea, Seoul, Korea) and methanol (Fisher Scientific Korea) used in the analysis were HPLC grade and ACS grade was used.

### Statistical analysis

All data are presented as the mean ± standard error of the mean (SEM). Significant differences between vehicle control and experimental values were calculated by one-way analysis of variance (ANOVA) followed by Student’s t-tests. All analyses were performed with Sigma Plot (Systat Software Inc., San Jose, CA, USA). Significant values are denoted by an asterisk (^*^P < 0.05 and ^**^P < 0.01 compared to vehicle group).

## Supplementary information

Dataset 1.

## Data Availability

The datasets generated during and/or analysed during the current study are not publicly available due to patent registration but are available from the corresponding author on reasonable request.
