# Supplementary material for: Gastroprotective effect of phytoncide extract from Pinus koraiensis pinecone in Helicobacter pylori infection
Source: Sci Rep. 2020 Jun 12;10:9547. doi: 10.1038/s41598-020-66603-8 (PMC7293342; doi:10.1038/s41598-020-66603-8)
Supplement: Supplementary file 1 — Dataset 1. [file 41598_2020_66603_MOESM1_ESM.docx]

**Supplementary Information**

**Gastroprotective effect of phytoncide extract from *Pinus koraiensis* pinecone in *Helicobacter pylori* infection**

Se-eun Kim^3,a^, Azra Memon^1^, Bae Yong Kim^2,3^, Hyelin Jeon^1,a^, Woon Kyu Lee^1,*^, and Se Chan Kang^3,*^

^1^Department of Biomedical Sciences, School of Medicine, Inha University, Incheon 22212, South Korea

^2^Research Institute, Phyrus Co., LTD., Danyang-gun 27000, Chungcheongbuk-do, South Korea

^3^Department of Oriental Medicine Biotechnology, College of Life Sciences, Kyung Hee University, Yongin-si 17104, Gyeonggi-do, Korea

^a^ Se-eun Kim and Hyelin Jeon contributed equally to this work

**Table of Contents**

| S.N. | Contents | Page |
| --- | --- | --- |
| 1. | PCR Gel-doc images (G1 to G7) | S2-S5 |

**
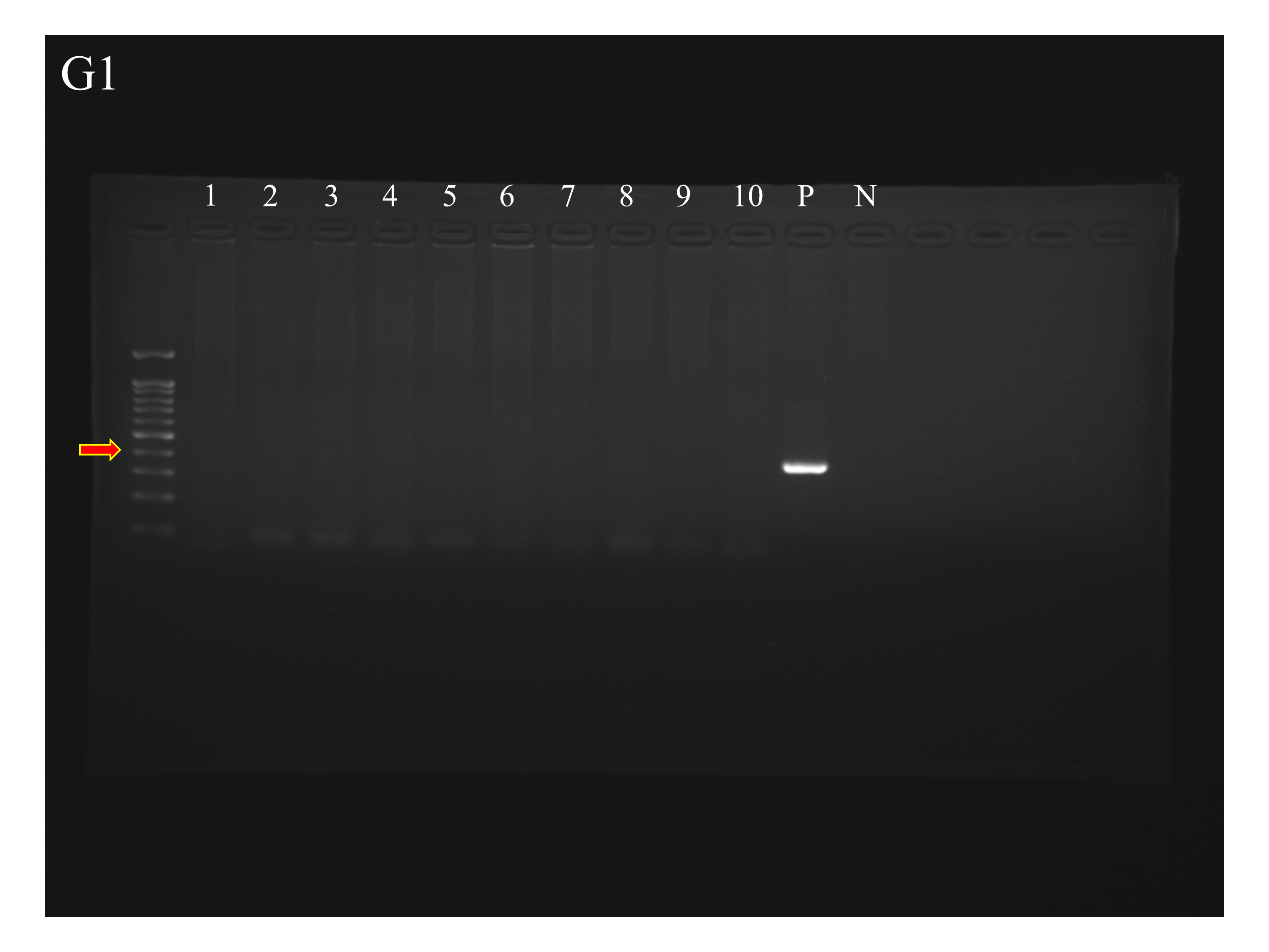
**

**
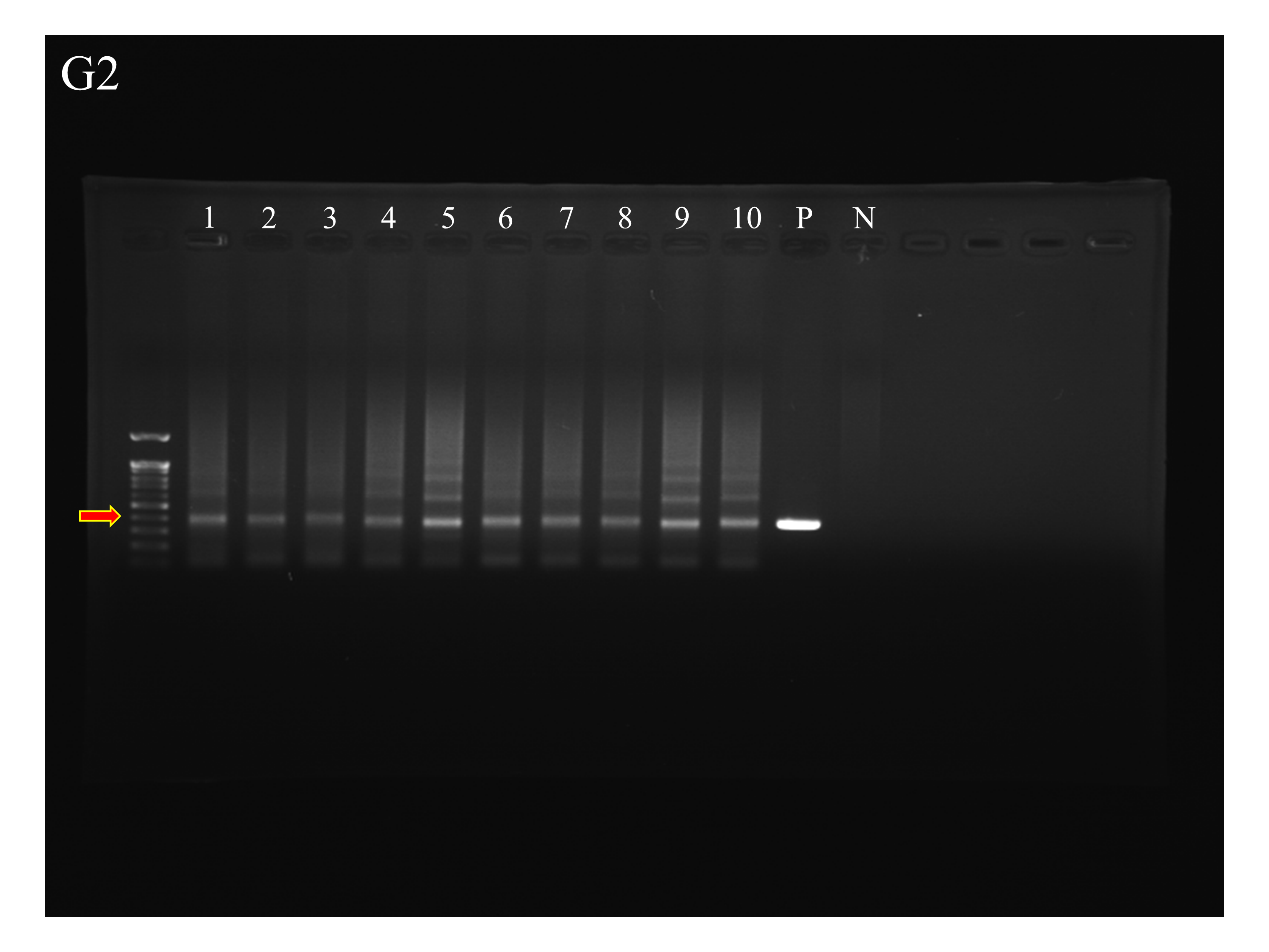

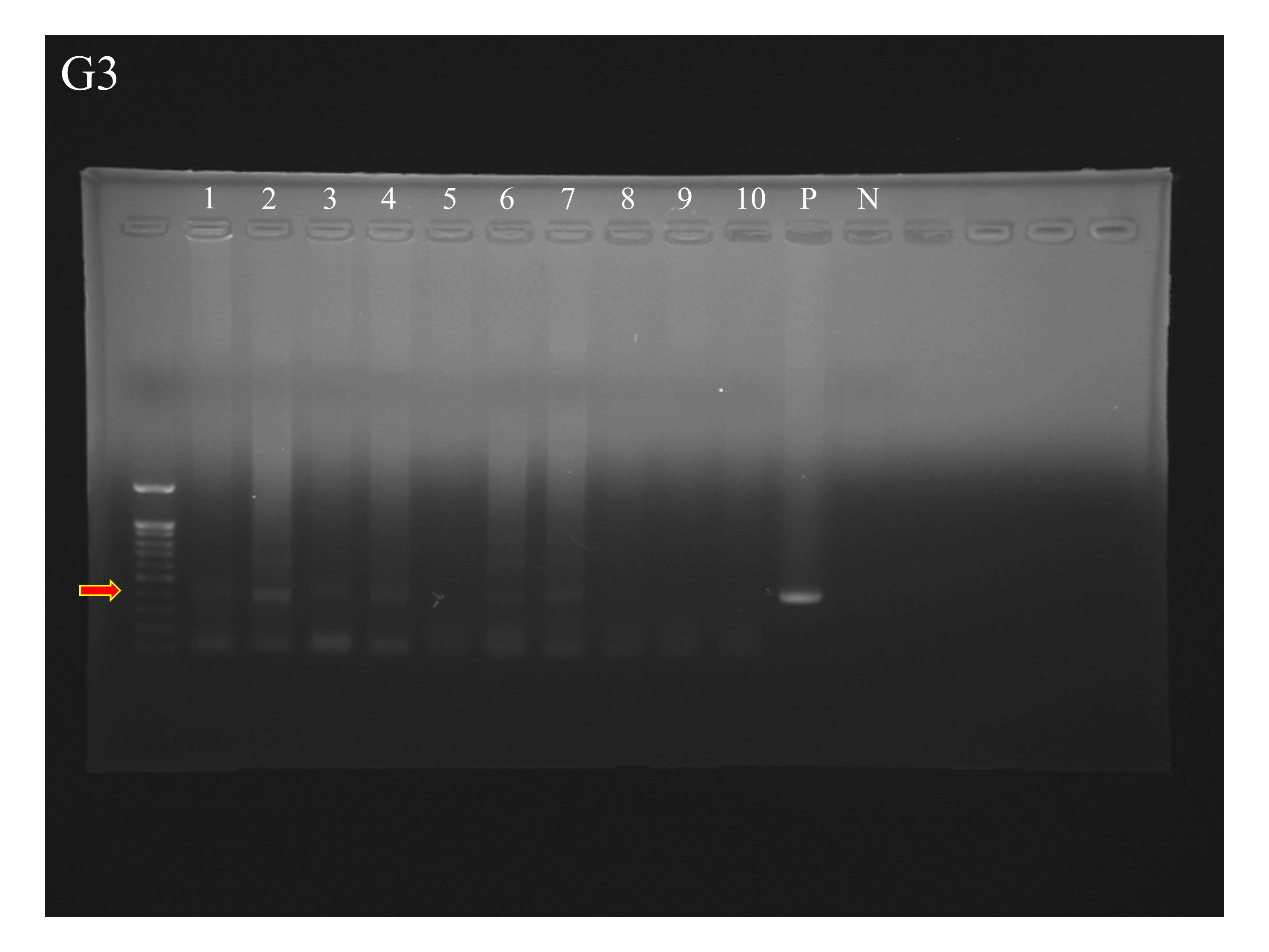

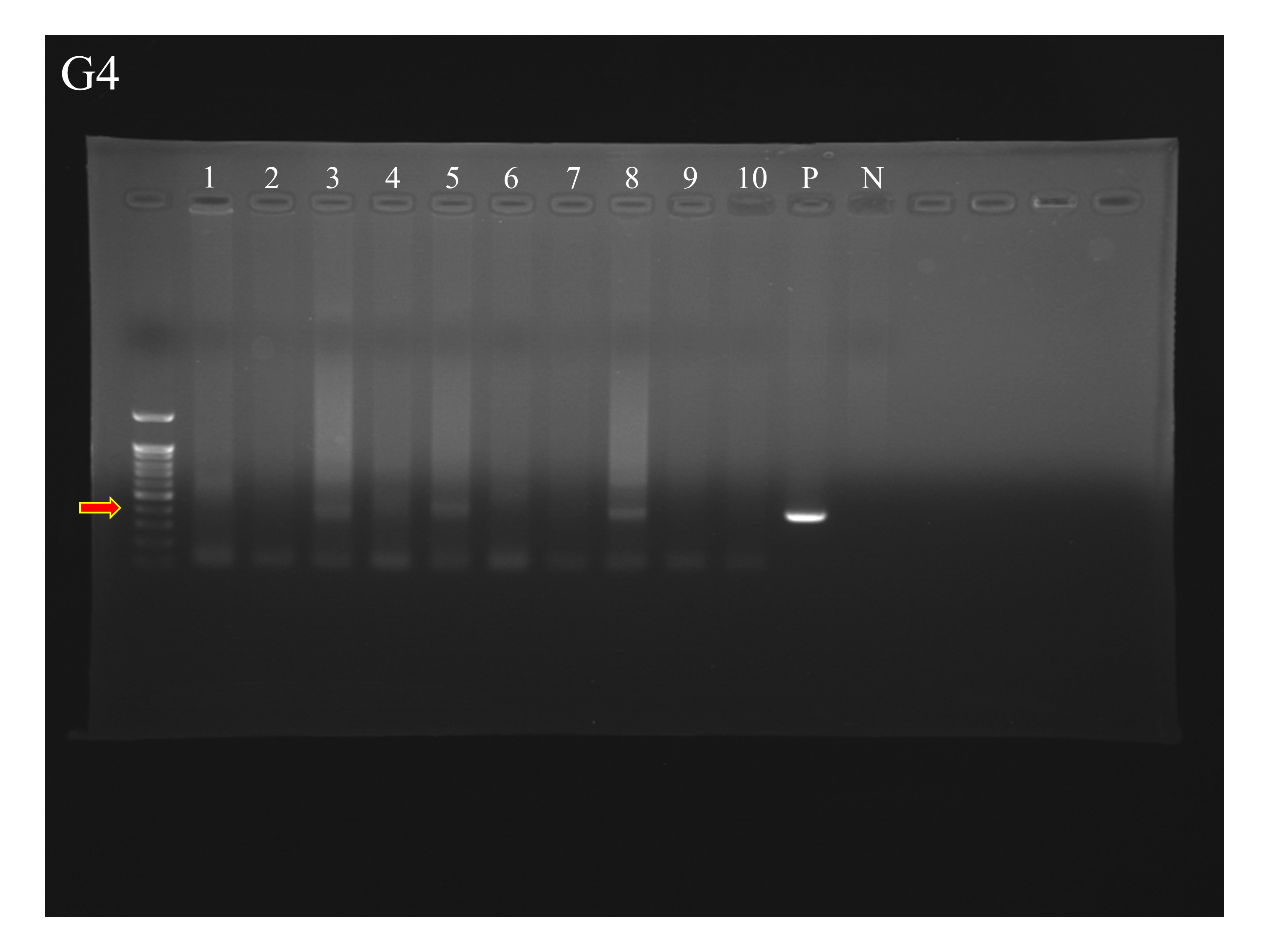

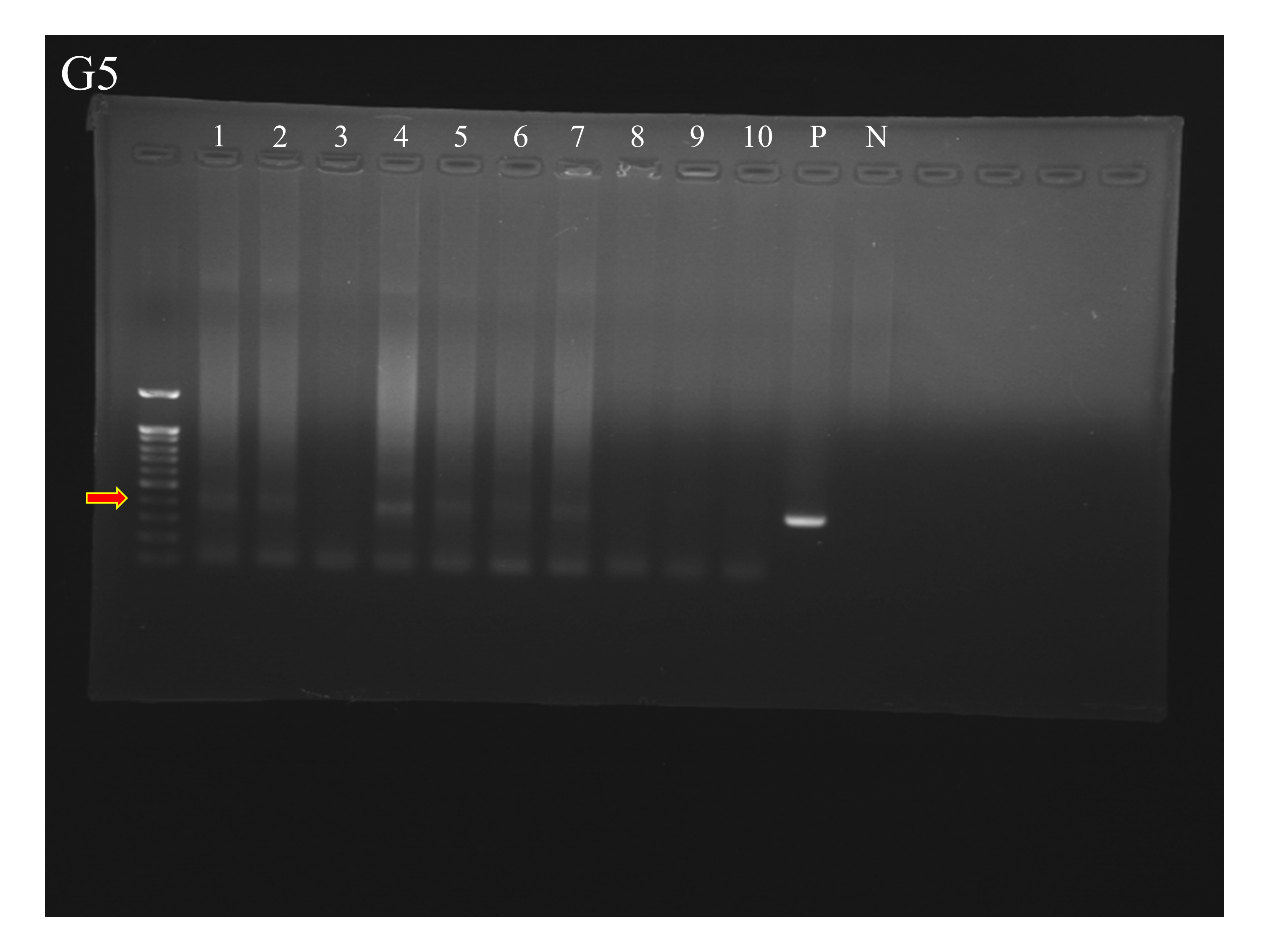

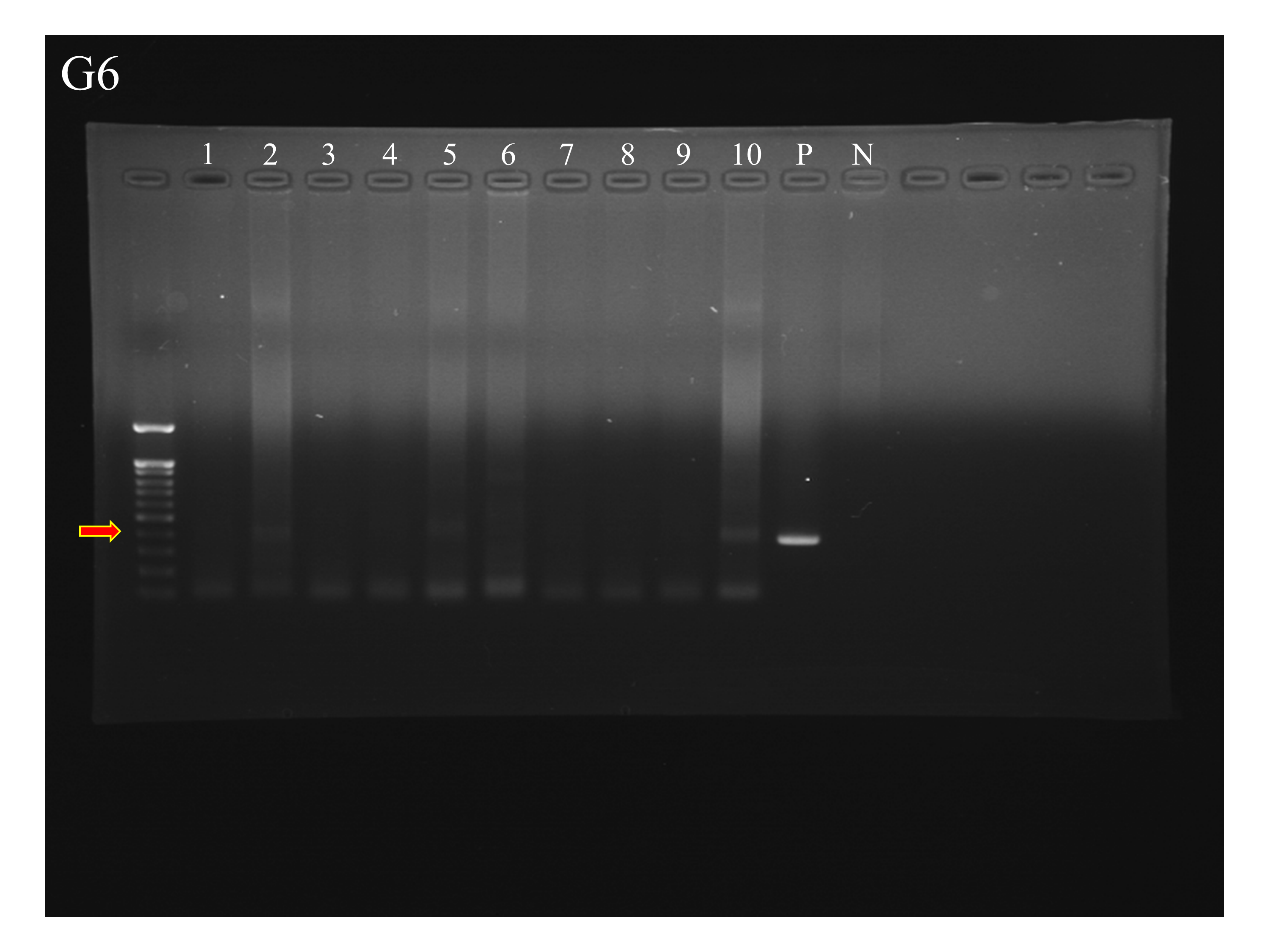
**

**
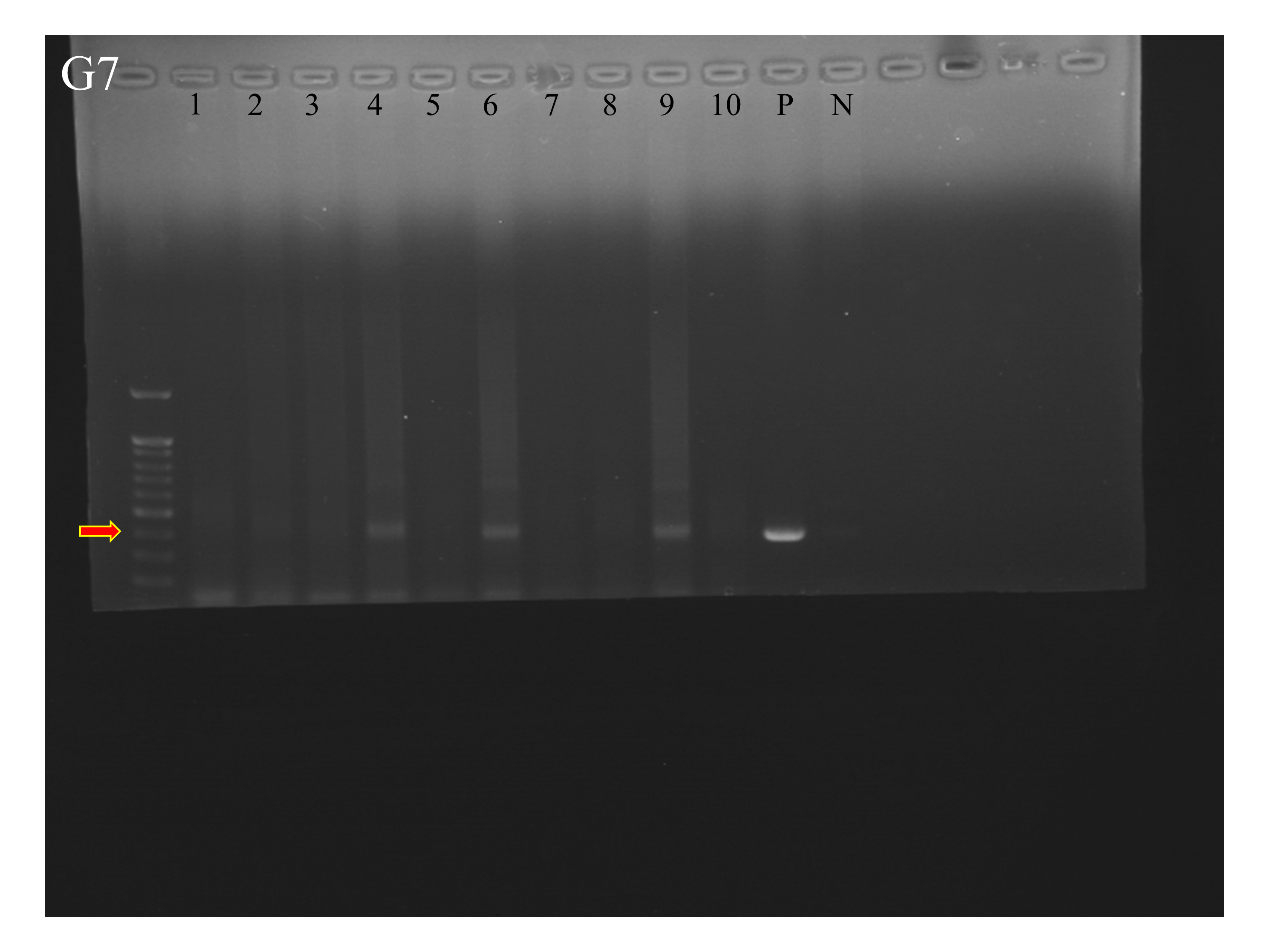
**

**Figure S1:** Raw data of cytotoxin gene (CagA) expression by PCR analysis

The results of the amplification of the CagA gene by extracting DNA from mouse gastric mucosa of each group (*n* = 10). The size of the amplified gene was 298 bp, and a band was identified near the fourth marker below. In the case of G1, the CagA gene was not detected in any of the lanes other than the H. pylori positive control lane because it was not infected with H. pylori. G2 was infected with H. pylori and there was no treatment, so a strong CagA band was detected in all mice. G3 and G4 are the results of providing the therapeutic drugs for H. pylori, and G5 to G7 are the results of providing the phytoncide.
